# Explaining parent-child (dis)agreement in generic and short stature-specific health-related quality of life reports: do family and social relationships matter?

**DOI:** 10.1186/s12955-016-0553-0

**Published:** 2016-10-21

**Authors:** Julia Quitmann, Anja Rohenkohl, Rachel Sommer, Monika Bullinger, Neuza Silva

**Affiliations:** 1 Department of Medical Psychology, University Medical Center Hamburg-Eppendorf, Martinistraße 52 D, 20246 Hamburg, Germany; 2 Cognitive and Behavioural Center for Research and Intervention, Faculty of Psychology and Educational Sciences of the University of Coimbra, Rua do Colégio Novo, 3001-802 Coimbra, Portugal

**Keywords:** Quality of life, Extent and direction of parent-child discrepancies, Parent-child dyadic approach, Parental burden, Growth hormone deficiency, Idiopathic short stature, Social support

## Abstract

**Background:**

In the context of health-related quality of life (HrQoL) assessment in pediatric short stature, the present study aimed to examine the levels of agreement/disagreement between parents’ and children’s reports of generic and condition-specific HrQoL, and to identify socio-demographic, clinical and psychosocial variables associated with the extent and direction of parent-child discrepancies.

**Methods:**

This study was part of the retest phase of the QoLISSY project, which was a multicenter study conducted simultaneously in France, Germany, Spain, Sweden and UK. The sample comprised 137 dyads of children/adolescents between 8 and 18 years of age, diagnosed with growth hormone deficiency (GHD) or idiopathic short stature (ISS), and one of their parents. The participants completed child- and parent-reported questionnaires on generic (KIDSCREEN-10 Index) and condition-specific HrQoL (QoLISSY Core Module). Children/adolescents also reported on social support (Oslo 3-items Social Support Scale) and parents assessed the parent-child relationships (Parental Role subscale of the Social Adjustment Scale) and burden of short stature on parents (QoLISSY- additional module).

**Results:**

The parent-child agreement on reported HrQoL was strong (intraclass correlation coefficients between .59 and .80). The rates of parent-child discrepancies were 61.5 % for generic and 35.2 % for condition-specific HrQoL, with the parents being more prone to report lower generic (42.3 %) and condition-specific HrQoL (23.7 %) than their children. The extent of discrepancies was better explained by family and social relationships than by clinical and socio-demographic variables: poorer parent-child relationships and better children’s social support were associated with larger discrepancies in generic HrQoL, while more parental burden was associated with larger discrepancies in condition-specific HrQoL reports. Regarding the direction of discrepancies, higher parental burden was significantly associated with parents’ underrating, and better children’s social support was significantly associated with parents’ overrating of condition-specific HrQoL.

**Conclusions:**

Routine assessment of pediatric HrQoL in healthcare and research contexts should include child- and parent-reported data as complementary sources of information, and also consider the family and social context.

## Background

Short stature is a chronic health condition statistically defined as a body height of two or more standard deviations (SD) below the mean for age and gender specific norms [1]. The diagnosis of growth hormone deficiency (GHD) in children requires a comprehensive clinical and auxological assessment, combined with biochemical tests and radiological evaluation [2]. However, GHD represents only 5 % of cases [3] and alternative causes of short stature and low growth velocity for age and pubertal stage need to be also considered and excluded. One of the normal variants is idiopathic short stature (ISS), a group of short children with sufficient growth hormone (GH) secretion, normal birth size and no evidence of systemic disease, psychiatric disorders or malnutrition [4]. The psychosocial consequences of short stature are well documented in the literature, but findings across studies are frequently contradictory [5]. While some studies have described significant health-related quality of life (HrQoL) impairment and more psychological problems among children and adolescents with GHD or ISS [6, 7], other studies have failed to detect differences from population norms [8]. The inconsistent results across studies can be attributed to methodological issues, namely the sources of information and the measures used to assess health outcomes. Parents of children with short stature tend to rate their children as having lower social functioning, poorer self-esteem, and more behavioral and cognitive problems than children with average height, while this view is rarely shared by young patients [9, 10]. This limited parent-child agreement calls for capturing both patients’ and parents’ reports in order to better understand the impact of short stature on children’s wellbeing and functioning [11].

Over the past years, a number of cross-culturally validated instruments have been developed to assess HrQoL in children and adolescents because of the growing interest on the perception and evaluation of an individual’s own life from a subjective perspective. The value of obtaining children’s self-reports about their health, functioning, abilities, and emotions is increasingly recognized within both medical care and child health research [12] and, thus, self-assessment of HrQoL is generally preferable to observer assessments. However, this is only possible for children and adolescents who are capable of providing the necessary information as a result of their age, their cognitive development, language skills and their health state [13]. Therefore, the majority of pediatric HrQoL instruments have been developed for children above 8 years of age and observer reports (usually a parent) have been used to gain information about younger children [14]. Several generic and disease-specific HrQoL measures that include parallel child- and parent-report versions are currently available, such as the generic KIDSCREEN [15], the chronic-generic DISABKIDS [16] and the short stature specific Quality of Life in Short Stature Youth (QoLISSY) [17].

There is considerable debate about the value of observational assessments (by clinicians or parents) and it has been argued that children and adolescents may operate within different reference systems and thus differ from adults in their understanding of HrQoL [13]. While parents can easily identify behavioral problems, this may not be the case with emotional problems such as sadness or tension [18]. Parents often lack first-hand information, especially regarding their child’s social functioning. Verhey and colleagues [19] also stressed that private feelings experienced by children and youth with a chronic disease and their desire to keep these experiences as a secret means that parents may be unaware of the nonvisible experiences and non-expressed feelings of their children. Still, parent-reports provide important complementary information about children’s HrQoL [20], especially since parents have more developed cognitive capabilities and life experiences than their children, enabling them to think about the future concerns of their child and how they might adapt to life with a chronic health condition later in life. Specifically in pediatric short stature, parental concerns about their child’s physical health and psychosocial well-being have been reported as important factors that contribute to the increase of both referrals by primary care providers to specialists and prescriptions of GH by endocrinologists, regardless of objective measures of the child’s growth [21–23].

The availability of measures with parallel child and parent versions has raised questions about the level of agreement between children’s own views and those of their parents [12]. It has been argued that discrepancies between child- and parent-reports could validly reflect each respondent’s perspective and they are not merely due to inaccuracy or bias [24] and that a number of studies and reviews in recent years have examined parent-child (dis)agreement [13, 25]. However, the literature is relatively incosistent, with reports of poor/moderate to high parent-child agreement. In their review about child and parent quality of life (QoL) ratings, Eiser and Morse [13] concluded that agreement is dependent on the domain being measured, with higher levels of agreement for physical aspects of health compared to emotional or social dimensions. There is also evidence of higher agreement between parents and their chronically ill children compared to parents and their healthy children [26]. While parents, in general, tend to overestimate their healthy child’s HrQoL [12, 26–28], parents of chronically ill children tend to rate their child’s HrQoL lower than the children do themselves [29, 30].

Agreement between child- and parent-ratings may also vary by the age of the child. Although some studies have described greater agreement between parents and younger children, suggesting that increasing independence in adolescence may limit the sharing of experiences with parents [31, 32], other studies have reported that younger age predicts greater differences between parents and children [33, 34]. An additional factor for consideration here is the impact of parents’ own functioning and well-being. Eiser et al. [35] found that mothers who rated their own well-being as poor also rated their child’s QoL as poor, suggesting that parents project their own feelings in the judgments about the child’s functioning. These findings were supported by a study from Davis et al. [36], who highlighted the importance of assessing maternal mental health when measuring parent-reported HrQoL. In addition, Goldbeck and Melches [37] reported a significant interaction effect of parental QoL and patients’ self-reported QoL in predicting parental reports of their children’s QoL. Another study, which examined the factors influencing agreement between child self-reports and parent observer-reports [38] also showed that parent-child agreement in healthy populations can be affected by the parents’ own QoL, the domains being investigated and the children’s age.

Verhey et al. [19] described agreement between parent observer-reports and child self-reports as a function of the measure of concreteness, visibility, and externality of the variable being measured, which applies when QoL is conceptualized as a functional and objective phenomenon that incorporates factors such as functional impairment, emotional health, social activity, and cognitive functioning. When incorporating internal factors, such as self-perception in relationships and experience of social support into the measurement of QoL, agreement is less likely because parents’ psychosocial stressors and burden of their child’s disease could also negatively affect the parent-child communication and, thus, the extent to which a parent is able to comment on the child’s QoL. Another possible reason for the disagreement is that children with chronic diseases may be more prone to focus on and recall the positive experiences because they are engaged in a quest for normality, whereas parents tend to report negative perceptions in the life of their child in addition to positive ones, perhaps reflecting their worries about the future [39].

Due to these inconsistent findings and the fact that Brütt et al. [40] identified just seven generic and only five condition-specific instruments to examine the QoL of short statured children and adolescents, the aim of this study was to extend knowledge of the factors influencing child-parent (dis)agreement in short stature. Specific objectives were to examine the levels of (dis)agreement between parents’ and children’s reports of generic and condition-specific HrQoL, and to identify socio-demographic, clinical and psychosocial variables associated with the extent and direction of parent-child discrepancies, in a sample of 137 dyads of parents and their children diagnosed with GHD or ISS.

## Methods

### Participants and procedures

This study was part of the European Quality of Life in Short Stature Youth (QoLISSY) project, which was a multicenter study conducted simultaneously in five European countries (France, Germany, Spain, Sweden and UK) with the objective of developing a condition-specific HrQoL instrument for short statured children and adolescents between 8 and 18 years of age, as well as for parents of children aged 4–18 years-old [17]. According to standardized guidelines, the development of the QoLISSY instrument was carried out in three stages: (1) focus-groups with item generation, (2) pilot-test with cognitive debriefing, and (3) field test with retest [41]. The QoLISSY project was approved by the Ethics Committees at the pediatric endocrine centers where patients were recruited, as required by national regulation of the participating countries (namely, die Ethikkommission der Ärztekammer Hamburg, Deutschland; the METC; Onderwijscoordinatie & projecten in 1201 DA Hilversum; the Regionala etikprövningsnämnden I Göteburg, the Universitair Ziekenhuis Brussel, Commissie Medische Ethiek, the Comité d’ethique de Toulouse, France; the research ethics committee, NHS Lothian, Edinburgh, and the CEIC Hospital Clínic de Barcelona). Data collection and analysis were performed following data protection requirements of the European Parliament (Directive 95/46/EC of the European Parliament and of the Council of 24 October 1995 on the protection of individuals with regard to the processing and free movement of personal data).

In each of the collaborating countries, eligible patients were identified by the pediatric endocrinologists based on medical records. Detailed information about the study aims and procedures was provided in each respective language when the families attended to the endocrine centers for regular appointments. Informed consent was obtained from parents, together with informal assent from children/adolescents, as well as permission to extract medical data from the clinical records through their physicians. For the families who agreed to participate, the questionnaires that were to be independently completed by patients and parents were delivered by hand or sent by mail, together with a pre-stamped envelope for returning the completed questionnaires to the respective center. With the aim to receive data from about 50 % of the sample to examine test-retest reliability of assessment instruments, subjects who agreed to participate in the retest phase received a second mail about 2 weeks later. Additional measures were included in the retest to identify potential psychosocial determinants and components of HrQoL. Anonymized data were entered into a project specific SPSS database in each center, which included the computation of the height deviation at the time of assessment with reference to the national norms for age and gender, and was subsequently sent to the German coordinating center.

The current analyses use data from the participants in both field test and retest phases of the QoLISSY project. To be included in the sample, children and adolescents had to meet the following criteria: age between 8 and 18 years-old; clinical diagnosis of GHD or ISS; height below -2 SD from the norms for their age, gender and nationality, at the time of diagnosis; absence of defined comorbid chronic health conditions; and cognitive ability to understand and complete the questionnaires. One of the parents of the child/adolescent was included, as were parents of children aged 4–7 years (not included in the current analyses). In the field test phase, a total of 544 questionnaires were sent out to families and 337 (61.95 %) were returned to the growth clinics. The response rate across countries ranged from 48 to 92 %. Twelve questionnaires were excluded due to missing values in a ratio greater than 25 % of the values, totaling 325 participants in the field test. Of these, 165 participants also returned the retest questionnaires, representing about 50 % of the baseline sample, as planned. After subtracting the 28 patients aged between 4 and 7 years (because only parent-reports were included for this age group), the sample for the present study included 137 dyads of children/adolescents between 8 and 18 years of age, diagnosed with GHD or ISS, and one of their parents. The socio-demographic and clinical characteristics of the sample are presented in Table 1.

**Table 1:** Socio-demographic and clinical characteristic of the sample

		Children (*n* = 137)	Parents (*n* = 137)
Sociodemographic characteristics			
Age (in years), *M* (*SD*)		13.30 (2.74)	43.88 (5.15)
Age group, *n* (%)	Children 8–12 years	59 (43.1 %)	
	Adolescents 13–18 years	78 (56.9 %)	
Sex, *n* (%)	Male	68 (49.6 %)	17 (12.4 %)
	Female	56 (40.9 %)	104 (75.9 %)
	*Missing*	13 (9.5 %)	16 (11.7 %)
Country, *n* (%)	Sweden	41 (29.9 %)	
	Germany	41 (29.9 %)	
	France	24 (17.5 %)	
	UK	16 (11.7 %)	
	Spain	15 (10.9 %)	
Clinical characteristics			
Diagnosis, *n* (%)	GHD	60 (43.8 %)	
	ISS	77 (56.2 %)	
Treatment status, *n* (%)	GH treatment	73 (53.3 %)	
	Untreated	51 (37.2 %)	
	*Missing*	13 (9.5 %)	
Height deviation, *n* (%)	Above –2 SD (achieved normal height)	24 (17.5 %)	
	Below –2 SD (current short stature)	71 (51.8 %)	
	*Missing*	42 (30.7 %)	

### Variables and measures

#### Health-related quality of life

The children’s and adolescents’ HrQoL were measured at the generic and condition-specific levels by the KIDSCREEN-10 Index and the core module of the QoLISSY questionnaire, respectively. The KIDSCREEN-10 Index [42] is a generic one-dimensional questionnaire which contains 10 items measuring physical well-being (e.g., “Have you felt fit and well?”), psychological well-being (e.g., “Have you felt sad?”), parent relations and autonomy (e.g., “Have your parent(s) treated you fairly?”), social support and peers (e.g., “Have you had fun with your friends?”), and school environment (e.g., “Have you got on well at school?”), with parallel forms to be answered by 8–18 year-old children/adolescents and by their parents/caregivers. The QoLISSY questionnaire is a condition-specific instrument targeting patient-reported HrQoL of 8–18 year-old children/adolescents with short stature, as well as the parent-reported HrQoL of 4–18 year-old patients [17]. Its core module consists of 22 items, assessing three HrQoL domains: Physical (six items measuring the physical limitations in everyday life due to short stature; e.g., “My height prevents me from doing things that other children my age do.”), Social (eight items referring to the way short stature interferes with the child’s social life; e.g., “Because of my height I get laughed at or teased.”), and Emotional (eight items assessing the child’s feelings and emotions with regards to his/her short stature; e.g., “Because of my height I feel different from others my age.”). Both questionnaires were answered using a five-point Likert scale ranging from 1 (*never*/*not at all*) to 5 (*always*/*extremely*). Standardized scores ranging from 0 to 100 were calculated for the KIDSCREEN index and for the QoLISSY Physical, Social and Emotional domains and total score, with higher scores indicating better HrQoL. Both child- and parent-report forms demonstrated good reliability in the current sample (see Table 2).

**Table 2:** Descriptive statistics, intraclass correlation coefficients, ANCOVA for repeated measures, and absolute and directional discrepancies

	Parent-report		Child-report			ANCOVA for repeated measures ^b^		Discrepancy		ANCOVA for repeated measures ^e^			
								Absolute ^c^	Directional ^d^	Absolute discrepancy		Directional discrepancy	
Baseline	*M* (*SD*)	*α*	*M* (*SD*)	α	ICC ^a^	*F*	*p*	*M* (*SD*)	*M* (*SD*)				
KIDSCREEN-10	73.15 (13.10)	.83	77.02 (14.02)	.84	.65	0.31	.58	9.15 (6.77)	−4.07 (10.66)				
QoLISSY-22	71.09 (22.63)	.95	75.34 (20.66)	.93	.75	2.28	.14	11.49 (11.46)	−1.02 (16.23)				
Physical QoL	73.67 (20.99)	.83	73.76 (20.29)	.77	.68	0.03	.87	12.73 (12.38)	−2.91 (17.55)				
Social QoL	71.52 (27.14)	.93	76.31 (22.65)	.87	.73	5.32	.02	13.97 (13.06)	−5.58 (18.33)				
Emotional QoL	68.72 (23.54)	.86	75.57 (24.27)	.85	.66	1.16	.29	10.86 (10.11)	−3.22 (14.51)				
Retest	*M* (*SD*)	*α*	*M* (*SD*)	α	ICC ^a^	*F*	*p*	*M* (*SD*)	*M* (*SD*)	*F*	*p*	*F*	*p*
KIDSCREEN-10	76.43 (12.69)	.82	78.99 (15.74)	.88	.59	2.58	.11	9.77 (7.87)	−3.77 (11.99)	0.28	.60	0.70	.41
QoLISSY-22	70.86 (22.39)	.95	73.81 (22.77)	.95	.80	2.52	.12	10.44 (10.29)	−0.84 (14.66)	2.16	.15	0.11	.74
Physical QoL	72.42 (21.49)	.83	73.04 (21.58)	.81	.77	0.04	.85	11.54 (11.97)	−2.79 (16.42)	0.39	.54	0.01	.91
Social QoL	70.93 (25.49)	.91	73.93 (25.23)	.89	.77	4.49	.04	12.63 (12.03)	−4.55 (16.87)	0.45	.51	0.03	.86
Emotional QoL	69.61 (24.19)	.87	74.28 (24.83)	.87	.72	2.35	.13	9.63 (10.12)	−2.90 (13.69)	0.78	.38	0.01	.93

^a^ Intraclass correlation coefficients reference values: ICC < .40 = poor agreement, ICC between .41 and .60 = moderate agreement, ICC between .61 and .80 = good agreement, ICC > .81 = excellent agreement (Landis & Koch, 1977). All ICCs were statistically significant at the .01 level

^b^ Univariate analyses of covariance for repeated measures, entering the informant (parent vs. child) as the within-subject factor and the socio-demographic and clinical variables (children’s sex and age group, diagnosis, treatment status and height deviation) as covariates, at baseline and retest

^c^ Absolute discrepancy = Σ (|parent score - child score|)/number of items for each dimension

^d^ Directional discrepancy = Σ (parent score - child score)/number of items for each dimension

^e^ Univariate analyses of covariance for repeated measures, entering the time of assessment (baseline vs. retest) as the within-subject factor and the socio-demographic and clinical variables (children’s sex and age group, diagnosis, treatment status and height deviation) as covariates

#### Family and social relationships

Parent-child relationships were assessed by both family members-the child and the parent-with the Parental Role scale of the Social Adjustment Scale-Self Report (SAS-SR) [43]. The SAS-SR is a 42-item questionnaire, measuring instrumental and expressive role performance over the past 2 weeks in six major areas of social functioning: work (either as a paid worker, homemaker, or student); social and leisure activities; relationships with extended family; role as a marital partner; parental role; and role within the family unit, including perceptions about economic functioning. The questions within the parental role area cover four categories: lack of involvement (e.g., “Have you been interested in what your children are doing-school, play or hobbies during the last 2 weeks?”), impaired communication (e.g., “Have you been able to talk and listen to your children during the last 2 weeks?”), friction (e.g., “How have you been getting along with the children during the last 2 weeks?”) and lack of affection (e.g., “How have you felt toward your children these last 2 weeks?”). Each item was rated on a five-point scale and a total score for the parental role area was calculated by summing up the scores of all the items within that area, with higher scores indicating more adjusted parent-child relationships. In the current sample, the Cronbach’s alpha value was .83 for child-reports and .77 for parent-reports, attesting the adequate reliability of the measure.

Social support was assessed by the children/adolescents using the Oslo 3-items Social Support Scale (OSS-3) [44], which is a brief measure composed of three items assessing the number of close confidants (“How many people are so close to you that you can count on them if you have serious problems?”), sense of concern or interest from other people (“How much concern do people show in what you are doing?”), and relationship to neighbors (“How easy can you get practical help from neighbors if you should need it?”). Although the response categories are independent for each of the three questions, a total score can be obtained for the OSS-3 by adding up the raw scores (range between 3 and 14 points), with higher scores indicating stronger perceived social support. The low Cronbach’s alpha coefficients found in the original study (α = .60) and in the current sample (α = .44) may reflect the multidimensionality of the social support construct, including its structural (quantitative dimension related to the amount of people in the individual’s social network and the amount of interconnections between its members) and functional dimensions (qualitative dimension referring to the emotional, instrumental and informational resources provided by social interactions) [45]. Despite the low reliability of the social support composite index, the feasibility of the OSS-3 and its predictive validity regarding psychosocial distress has been confirmed in several studies [46, 47].

Finally, the burden of the child’s short stature on the parents was assessed with the Effects on Parents scale of the QoLISSY questionnaire [17]. This scale is embedded in the parent-report version and was developed as a complementary scale for assessing potential determinants of pediatric HrQoL (along with Coping, Height-related Beliefs, Treatment Experiences and Concerns about the child’s Future scales, which were not included in the present study). The Effects on Parents scale consists of 11 items (e.g., “My child’s growth problems make me feel anxious.”) scored on a 5-point Likert scale ranging from 1 (*never*/*not at all*) to 5 (*always*/*extremely*) and providing a 0–100 standardized score, with higher scores indicating less negative effects of the child’s condition on the parents. In the current sample, the scale presented very good reliability, with a Cronbach’s alpha value of .92.

#### Socio-demographic and clinical data

The socio-demographic data included patients’ and parents’ sex, date of birth and nationality. Physician-reported clinical data included diagnosis (GHD or ISS), treatment status and height at time of diagnosis and at time of assessment. The treated group consisted of patients receiving rhGH treatment at the time of assessment and before; the untreated group had never been treated. Although a height deviation greater than -2SD at time of diagnosis was required for patient inclusion, some had an achieved height above -2SD at the time of assessment due to treatment. Thus, the current height deviation was categorized into two groups: achieved normal height (height deviation > -2 SD) and current short stature (height deviation ≤ -2 SD).

### Data analyses

Statistical analyses were performed with SPSS 20.0 (SPSS Inc., Chicago, IL, USA). Except for clinical and socio-demographic variables, missing data were handled by individual mean score allocation, if they were random and less than 25 % of the values. Descriptive statistics were calculated for socio-demographic and clinical variables.

The child-parent (dis)agreement on baseline and retest reports of HrQoL were examined at the individual and the group levels [48], by using, respectively, intraclass correlation coefficients [ICC] (two-way mixed model, absolute agreement, 95 % confidence interval [CI]) and analyses of covariance for repeated measures. We performed univariate analyses of covariance for the total scores on generic KIDSCREEN and condition-specific QoLISSY measures, and multivariate analyses of covariance for the three core dimensions of condition-specific HrQoL (i.e., physical, social and emotional), entering the informant (parent vs. child) as the within-subject factor and the socio-demographic and clinical variables (children’s sex and age group, diagnosis, treatment status and height deviation) as covariates.

Absolute discrepancies (the mean of the absolute differences between the reports from the parent and the child) and directional discrepancies (the mean of the parent-child differences) were computed as dyadic indexes of the extent and direction of disagreement, respectively [49]. Directional discrepancies were categorized into three groups (“parent-report < child-report”, “agreement” and “parent-report > child-report”) based on the threshold for clinically important differences in quality of life [50]. Thus, agreement was defined as an absolute difference between the parent’s and the child’s scores that were lower than or equal to half of the standard deviation (SD) of the score with the greatest variability. Differences in absolute and directional parent-child discrepancies from the baseline to the retest were examined with univariate analyses of covariance for the total scores on KIDSCREEN and QoLISSY measures, and multivariate analyses of covariance for the three core dimensions of condition-specific HrQoL, entering the time of assessment (baseline vs. retest) as the within-subject factor and the socio-demographic and clinical variables as covariates.

In order to identify the clinical, socio-demographic and psychosocial variables associated with the extent of parent-child discrepancies (absolute discrepancies), hierarchical multiple regression analyses were performed, entering the clinical and sociodemographic variables (i.e., children’s sex, age group, diagnosis, treatment status and height deviation) in the first block and the psychosocial variables (i.e., parent-child relationships as perceived by both family members, children’s social support and burden of the child’s short stature on the parents) in the second block of the regression equation. Multinomial logistic regressions, using “agreement” as the reference category [51], were performed to identify which variables were associated with the direction of the parent-child discrepancies (directional discrepancies). The clinical and sociodemographic variables were entered in the equation as categorical factors and the psychosocial variables as covariates. The goodness-of-fit of the overall model was evaluated using the likelihood ratio tests, as well as the Cox and Snell’s *R* ^2^ and Nagelkerke’s adjusted *R* ^2^ as indicators of effects sizes, considering *R* ^2^ > .10 as small effect, *R* ^2^ > .30 as medium effect, and *R* ^2^ > .50 as large effect [52]; and the statistical significance of individual predictors was evaluated by calculating the Wald statistic and the odds ratio (OR) with a 95 % CI.

## Results

### Parent-child (dis)agreement

At the individual level, there were strong intraclass correlations between children’s and parents’ reports of generic and condition-specific HrQoL, at both baseline and retest (Table 2). At the group level, the results indicated that the parents’ and children’s reports of generic and condition-specific HrQoL (total scores) were not significantly different, after controlling for socio-demographic and clinical variables. In addition, the multivariate effect of the informant on three core domains of condition-specific HrQoL did not reached statistical significance, Wilks’ Lambda = .92, *F*_(3, 84)_ = 2.46, *p* = .07 at baseline, and Wilks’ Lambda = .93, *F*_(3, 83)_ = 2.16, *p* = .10 at retest, even if the univariate analyses (Table 2) suggested that parents’ and children’s reports of social HrQoL were significantly different, with children rating their social HrQoL as higher than did the parents.

**Fig. 1:**
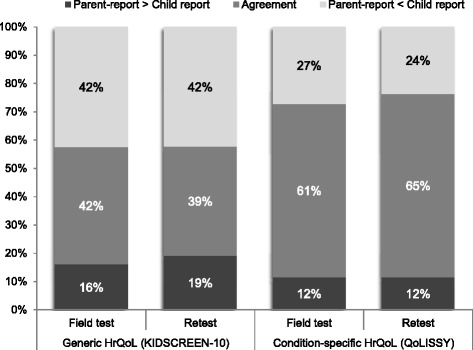
Distribution of parent-child directional discrepancies on reports of generic and condition-specific HrQoL total scores

As depicted in Fig. 1, the examination of the parent-child discrepancies indicated higher rates of agreement for the condition-specific measure (61.2 % at baseline and 64.9 % at retest) than for the generic HrQoL assessment (41.5 % at baseline and 38.5 % at retest). In cases where disagreement occurred, parents were more likely to rate both generic and condition-specific HrQoL lower than did the children themselves. The multivariate analyses of covariance for repeated measures indicated no significant differences in the extent, Wilks’ Lambda = .96, *F*_(3, 82)_ = 1.05, *p* = .37, or direction of parent-child discrepancies, Wilks’ Lambda = 1.00, *F*_(3, 82)_ = 0.06, *p* = .98, from the baseline to the retest. The results for univariate analyses are presented in Table 2.

### Regression analyses explaining the extent of disagreement

The results from the hierarchical regression analyses conducted to identify the variables associated with the extent of the parent-child disagreement (Table 3) showed that the clinical and sociodemographic variables did not contribute significantly to explain the variance of absolute discrepancies in generic (Δ*R* ^2^ = .06; *F*_(5, 63)_ = 0.74, *p* = .59) and condition-specific (Δ*R* ^2^ = .13; *F*_(5, 64)_ = 1.88, *p* = .11) HrQoL reports. However, controlling for children’s sex, age group, diagnosis, treatment status and current height deviation, a significant proportion of the variance of parent-child absolute discrepancies was explained by the psychosocial variables (Δ*R* ^2^ = .20; *F*_(4, 59)_ = 3.89, *p* < .01 for generic HrQoL reports; Δ*R* ^2^ = .17; *F*_(4, 60)_ = 3.54, *p* = .01 for condition-specific HrQoL reports). Specifically, poorer parent-child relationships as perceived by the parents (β = -.39; *t* = -2.71, *p* < .01) and better children’s perceptions of social support (β = .30; *t* = 2.30, *p* = .03) were associated with larger discrepancies in generic HrQoL reports, while greater parental burden was associated with larger discrepancies in condition-specific HrQoL (β = -.42; *t* = -3.28, *p* < .01).

**Table 3:** Hierarchical regression model explaining the extent of parent-child discrepancies

	Absolute parent-child discrepancies			
	Generic HrQoL *R* ^2^ = .25; *F*_(9, 59)_ = 2.22^*^		Condition-specific HrQoL *R* ^2^ = .29; *F*_(9, 60)_ = 2.78^**^	
*First step:*	∆*R* ^2^ = .06; *F*_(5, 63)_ = 0.74		∆*R* ^2^ = .13; *F*_(5, 64)_ = 1.88	
*Sociodemographic and clinical variables*	*B* (SE)	*t*	*B* (SE)	*t*
Children’s sex ^a^	0.48 (1.83)	0.26	−0.99 (2.37)	−0.42
Children’s age group ^b^	−2.12 (2.07)	−1.03	−2.29 (2.69)	−0.85
Diagnosis ^c^	−4.10 (2.20)	−1.87	1.74 (2.87)	0.61
GH treatment ^d^	2.67 (3.00)	0.89	6.15 (3.78)	1.63
Current height deviation ^e^	−0.04 (2.04)	−0.02	−3.37 (2.66)	−1.27
*Second step:*	∆*R* ^2^ = .20; *F*_(4, 59)_ = 3.89^**^		∆*R* ^2^ = .17; *F*_(4, 60)_ = 3.54^*^	
*Psychosocial variables*	*B* (SE)	*t*	*B* (SE)	*t*
Children’s sex ^a^	−0.30 (1.76)	−0.17	−2.09 (2.30)	−0.91
Children’s age group ^b^	−1.08 (2.00)	−0.54	−0.48 (2.61)	−0.18
Diagnosis ^c^	−6.04 (2.24)	−2.70^**^	−2.49 (2.95)	−0.85
GH treatment ^d^	2.59 (2.77)	0.94	6.75 (3.52)	1.92
Current height deviation ^e^	−0.70 (2.02)	−0.35	−1.89 (2.64)	−0.72
parent-child relationships (child’s view)	0.27 (0.34)	0.79	0.01 (0.44)	0.01
parent-child relationships (parent’s view)	−1.19 (0.44)	−2.71^**^	−0.03 (0.58)	−0.05
Children’s social support	1.16 (0.50)	2.30^*^	1.12 (0.65)	1.71
Parental burden	−0.05 (0.33)	−1.48	−0.14 (0.04)	−3.28^**^

^**^ *p* < .01, two-tailed. ^*^ *p* < .05, two-tailed

^a^ Reference group: 0 = female; ^b^ Reference group: 0 = children 8–12 years-old; ^c^ Reference group: 0 = growth hormone deficiency; ^d^ Reference group: 0 = be receiving/have received GH treatment; ^e^ Reference group: 0 = height deviation above -2 SD (achieved normal height)

### Regression analyses explaining the direction of disagreement

The results from multinomial logistic regression analyses explaining the direction of parent-child disagreement (i.e., “child-report > parent-report” and “child-report < parent-report” versus “agreement”) are presented in Table 4. Although the overall model testing the clinical, sociodemographic and psychosocial predictors of directional discrepancies in generic HrQoL reports as assessed by the KIDSCREEN questionnaire (-2 Log-Likelihood = 114.21) was significantly better than the baseline model in which only the constant was included (-2 Log-Likelihood = 147.04; *χ* ^2^_(18)_ = 32.83, *p* = .02), no contribution of the individual predictors reached statistical significance. On the contrary, more burden of the child’s short stature on the parents was significantly associated with larger discrepancies in the direction of parents reporting worse condition-specific HrQoL than the children (Wald *χ* ^2^_(1)_ = 9.69, *p* < .01; OR = 0.96, 95 % CI: 0.93–0.98) and better children’s perceptions of social support were significantly associated with an increased likelihood of discrepancies in the direction of parents’ overrating pediatric HrQoL (Wald *χ* ^2^_(1)_ = 6.70, *p* = .01; OR = 2.43, 95 % CI: 1.24–4.75). The sociodemographic and clinical variables were not significantly associated with any direction of child-parent discrepancies in the QoLISSY questionnaire. The overall model testing the predictors of directional discrepancies in condition-specific HrQoL reports (-2 Log-Likelihood = 85.99) was significantly better than the baseline model in which only the constant was included (-2 Log-Likelihood = 118.80; *χ* ^2^_(18)_ = 32.81, *p* = .02), with a medium effect size (*R* ^2^_Cox_ & _Snell_ = .37; *R* ^2^_Nagelkerke_ = .46).

**Table 4:** Multinomial logistic regression model explaining the direction of parent-child discrepancies

	Directional parent-child discrepancies			
	Generic HrQoL *R* ^2^ = .38_(Cox_ & _Snell)_; .43 _(Nagelkerke)_ Model *χ* ^2^_(18)_ = 32.83^*^		Condition-specific HrQoL *R* ^2^ = .37_(Cox_ & _Snell)_; .46 _(Nagelkerke)_ Model *χ* ^2^_(18)_ = 32.81^*^	
*Child-report > Parent-report vs. Agreement*	*B* (SE)	OR (95 % CI)	*B* (SE)	OR (95 % CI)
Children’s sex ^a^	0.60 (0.70)	1.83 (0.47/7.16)	1.22 (0.78)	3.39 (0.76/15.20)
Children’s age group ^b^	1.38 (0.76)	3.97 (0.90/17.51)	0.46 (0.83)	1.58 (0.31/7.96)
Diagnosis ^c^	1.40 (0.92)	4.05 (0.67/24.64)	0.66 (0.95)	1.94 (0.30/12.35)
GH treatment ^d^	−1.11 (1.08)	0.33 (0.04/2.75)	0.43 (1.02)	1.54 (0.21/11.28)
Current height deviation ^e^	0.58 (0.73)	1.78 (0.42/7.49)	0.51 (0.76)	1.67 (0.37/7.46)
parent-child relationships (child’s view)	0.11 (0.13)	1.11 (0.86/1.44)	0.16 (0.14)	1.17 (0.89/1.53)
parent-child relationships (parent’s view)	−0.29 (0.18)	0.75 (0.53/1.07)	−0.23 (0.17)	0.79 (0.57/1.10)
Children’s social support	0.37 (0.21)	1.44 (0.96/2.15)	0.24 (0.22)	1.27 (0.82/1.97)
Parental burden	−0.01 (0.01)	0.99 (0.96/1.01)	−0.04 (0.01)	0.96 (0.93/0.98)^**^
*Child-report < Parent-report vs. Agreement*	*B* (SE)	OR (95 % CI)	*B* (SE)	OR (95 % CI)
Children’s sex ^a^	−1.44 (0.85)	0.24 (0.04/1.26)	−0.68 (1.38)	0.51 (0.03/7.54)
Children’s age group ^b^	−0.94 (1.02)	0.39 (0.05/2.89)	−1.00 (1.34)	0.37 (0.03/5.09)
Diagnosis ^c^	1.37 (1.08)	3.95 (0.47/32.87)	1.10 (1.63)	3.00 (0.12/73.57)
GH treatment ^d^	−1.65 (1.37)	0.19 (0.01/2.83)	−1.70 (1.90)	0.18 (0.01/7.57)
Current height deviation ^e^	−1.30 (1.07)	0.27 (0.03/2.24)	0.71 (1.40)	2.04 (0.12/31.67)
parent-child relationships (child’s view)	−0.21 (0.16)	0.81 (0.60/1.11)	0.08 (0.21)	1.08 (0.71/1.64)
parent-child relationships (parent’s view)	0.12 (0.23)	1.13 (0.72/1.76)	0.04 (0.33)	1.04 (0.55/1.97)
Children’s social support	0.13 (0.24)	1.14 (0.72/1.81)	0.89 (0.34)	2.43 (1.24/4.75)^**^
Parental burden	−0.01 (0.02)	0.99 (0.96/1.02)	0.01 (0.03)	1.00 (0.96/1.06)

^*^ *p* < .05, two-tailed. ^**^ *p* < .01, two-tailed

^a^ Reference group: 0 = female; ^b^ Reference group: 0 = children 8–12 years-old; ^c^ Reference group: 0 = growth hormone deficiency; ^d^ Reference group: 0 = be receiving/have received GH treatment; ^e^ Reference group: 0 = height deviation above -2 SD (achieved normal height)

## Discussion

To our knowledge, this is the first study examining the child-parent (dis)agreement in a sample of short statured children. The results of this study showed that agreement between child- and parent-reports can be affected by the domains investigated, and that the extent and direction of discrepancies were better explained by family and social relationships than by socio-demographic and clinical variables. Inconsistent with previous findings from the literature [13, 25], which advocate moderate levels of agreement in pediatric HrQoL assessment, we have found strong intraclass correlations between children’s and parents’ reports of generic and condition-specific HrQoL, with higher rates of agreement for the condition-specific QoLISSY questionnaire than for the generic KIDSCREEN questionnaire. Upton et al. [53] stated that the levels of agreement would depend on the relevance of different domains for a specific clinical group because parents would be most alert to the frailest domains of their children’s HrQoL. The greater level of agreement found for the short stature-specific measure may be explained by the relevance of the questions for this particular group of children and adolescents, diagnosed with GHD or ISS, and by the higher likelihood of parents to be more alert to stature-related issues than general aspects of HrQoL. When disagreement occurred, it was likely to be in the direction of parents reporting worse HrQoL than their children. The same pattern was found in other studies with children/adolescents with chronic health conditions [25, 54], but opposite to that observed in healthy populations. These results may reflect, on the one hand, the children’s tendency to emphasize the positive aspects of adaptation [55] and, on the other hand, the parents’ reliability in identifying the most strongly affected areas of their children’s functioning [56].

Surprisingly, the clinical and sociodemographic variables did not contribute significantly to explain the extent or direction of parent-child discrepancies. Previous research has also reported inconsistent findings regarding the children’s characteristics as factors influencing agreement rates, for example, Petsios and colleagues [57] found higher levels of agreement for older children supporting the hypothesis that the levels of agreement would depend on the cognitive and communication skills, while other studies [31, 32] described greater agreement between parents and younger children, suggesting that the development of independence during adolescence may limit the sharing of experiences with parents.

Conversely, the extent and direction of parent-child discrepancies on HrQoL reports were mainly explained by family- and social-related factors. Poorer parent-child relationships as perceived by the parents were associated with larger absolute discrepancies in generic HrQoL. This is important since the family context characterized by the parents’ ability to talk and listen to their child, by affection toward the child and actual interest in what the child is doing may allow children to openly express his/her worries and feelings. More than development-related communication skills, these parental attitudes may lead to an improved parental understanding of their child’s psychosocial functioning and decreasing discrepancies in HrQoL reports. It is also important to note that only the parents’ (and not the children’s) perceptions of parent-child relationships were significant predictors of absolute discrepancies; this may be explained by the parents’ greater awareness of their active efforts to listen and be attentive to their children’s needs.

Children’s perceptions of better social support were associated with larger absolute discrepancies in generic HrQoL and with larger discrepancies in the direction of parents reporting better condition-specific HrQoL than the children. These results suggest that a wide social network, in addition to parental support, can reduce parent-child communication and increase the discrepancy in HrQoL reports. This “negative” effect of social support in parent-child agreement may be explained by the child’s increased likelihood to share his/her experiences and feelings with several people he/she can rely on (e.g., extended family, neighbors, peers, etc.), other than the parents. However, this finding should not be interpreted as a “negative effect”, since it provides the child alternative ways of coping with the health condition and its psychosocial impairments, and may ease parents’ responsibilities and caregiving burden. A recent study in pediatric asthma found that a greater caregiving burden was associated with increased discrepancies in both directions [25]. By sharing caregiving responsibilities with the social support network and reducing the caregiving burden, the parents may become more likely to “normalize” the child’s health condition and rate their HrQoL as superior (nearing the pattern of parent-child discrepancies described for healthy children).

Moreover, greater parental burden resulting from caring for a child with short stature, including more worries and negative feelings, were associated with larger absolute discrepancies and with larger discrepancies in the direction of parents reporting worse condition-specific HrQoL than the children. White-Koning et al. [29] described a significant association between higher levels of parenting stress and parents’ underrating the HrQoL of their children with cerebral palsy. In addition, in another pediatric asthma study, the illness-related burdens experienced by parents were negatively associated with parents’ reports of pediatric HrQoL [33], which may contribute to child-parent disagreement in the direction of parents reporting worse HrQoL than the children. Parents’ perceptions of caregiving tasks and chronic-disease management routines as overly demanding and burdensome have also been associated with negative mother-child interactions [58], which may limit the exchange of information between children and parents. Moreover, the caregiving burden is likely to negatively affect parents’ perceptions of family relationships [59] and thus contribute indirectly to parent-child disagreement on HrQoL reports.

Some limitations should be acknowledged in the interpretation of the findings from the present study. Even if GHD and ISS are rare diseases and ISS is not routinely treated with GH in Europe, the small sample size may limit the generalizability of the results. In addition, the use of data only from the re-test phase of the European QoLISSY study and the exclusion of cases with missing clinical data, although random, may skew the data and reduce the statistical power of the analyses. Second, the non-probabilistic sampling strategy may have influenced the levels of agreement because parents who participated may be more involved in pediatric healthcare than parents who had refused to participate. In addition, most parents’ reports were provided by the mothers (75.9 %). Although this is common in pediatric research [60], the disproportionate participation of mothers and fathers limited the ability to assess the potential role of the parents’ sex in explaining the extent and direction of parent-child discrepancies. A third limitation concerns the methods of data collection. Since the questionnaires were completed by the parents and the children at home, a parental influence on children’s answers cannot be ruled out, even if the parents were specifically instructed to not interfere with their children’s responses. Despite the possibility of some bias introduced by any exchange of information between parent and child while they were filling in the questionnaires, we believe that the high levels of agreement would be better explained by the instrument characteristics (e.g., the relevance of different domains for this specific clinical group). Forth, the absence of psychosocial variables assessment at baseline prevents the examination of the impact of changes in family and social relationships in the rates of parent-child agreement. Finally, the low Cronbach’s alpha coefficient for the Oslo 3-items Social Support Scale must be mentioned.

## Conclusion

Despite the above mentioned limitations, our findings have important implications for research and clinical practice. Routine assessment of pediatric HrQoL in healthcare and research contexts should not only include self- and parent-reported data as complementary sources of information, they should also consider the family and social context. Besides, the possible (dis)agreement between parents and children should lead not only to the consideration of which informant is most objective or valid, but also to questions about what meaning these differences might have in the context of the parent-child relationship or the family’s psychosocial status and eventually lead to further assessment of these dimensions of the families’ life. The additional cost of conducting a more in-depth assessment of HrQoL and its determinants can be offset through a deeper understanding of how to interpret self- and observer-reported data. Based on knowledge about determinants of discrepancies in HrQoL ratings, prospective studies should address differences in scores as an option for integrating parent and patient reports in relation to clinical outcomes.

## Abbreviations

CI: Confidence interval
GH: Growth hormone
GHD: Growth hormone deficiency
HrQoL: Health-related quality of life
ICC: Intraclass correlation coefficient
ISS: Idiopathic short stature
OR: Odds ratio
OSS-3: Oslo 3-items social support scale
QoL: Quality of life
QoLISSY: Quality of life in short stature youth
SAS-SR: Social adjustment scale-self report
SD: Standard deviation
